# 

**Published:** 1903-08-22

**Authors:** 


### NOTICE TO CORRESPONDENTS.

All MSS., Letters, Books for Review, and other matters intended for the Editor should be addressed The Editor, "The Hospital," The Hospital Buildings, 28 & 29 Southampton Street, Strand, W.C.

The Editor cannot undertake to return rejected MS., even when accompanied by stamped directed envelope.

**Notice to Advertisers and Subscribers.**

All Advertisements, Orders for Copies of The Hospital, and Business Communications should be addressed to **THE MANAGER, (Not to the Editor)**, The Hospital, 28 & 29 Southampton Street, Strand, London, W.C.

The Cost of Subscription, post free, is as follows.—Per week, 3½d.; per quarter, 3s. 9d.; per half-year, 7s. 6d.; per annum, 15s. Foreign and Colonial:—Per annum, 19s. 6d., payable, in all cases, in advance.

**NOTICE.—Vol. XXXIII. of "The Hospital" (October 1902 to March 1903) in handsome cloth binding, will shortly be on sale at the office of this Journal, price 7s. 6d. Cases for binding the Numbers contained in this Volume 1s. 6d. Index to Vol. XXXIII., 2½d., post free.**

**The monthly part for August is now ready. Price 1s., by post 1s. 3d.**

### BOOKS RECEIVED.

THE SCIENTIFIC PRESS, LIMITED.

"Practical Guide to Surgical Bandaging and Dressings." By Dr. Johnson Smith.

"Nursing Notes on Midwifery and Gynæcology." By Sister Ross.

BAILLIÈRE, TINDALL AND COX.

"Aids to Infant Feeding." By John McCaw.

J. B. LIPPINCOTT CO.

"International Clinics." Vol. II. Thirteenth Series.

HEALTH DEPARTMENT, LIVERPOOL.

"Report on the Health of the City of Liverpool, 1902."

HEALTH RESORTS BUREAU.

"Health Resorts of Europe." By Dr. T. Linn.

KEGAN PAUL, TRENCH, TRUBNER AND CO.

"The Exact Science of Health based upon Life's Great Law." By Dr. R. Walter.

HODDER AND STOUGHTON.

"The Religious Sense in its Scientific Aspect." By Greville Macdonald.

### The Student of Medicine.

### APPOINTMENTS.

**Henry C. L. Birkbeck**, M.B., Medical Referee under the Workmen's Compensation Act for Bridgwater, Chard, Langport, Taunton, Wellington, and Williton, in County Court Circuit, No. 57. **R. T. Bruce**, M.B., M.S.Edin., Certifying Factory Surgeon for the Thame District, Oxford. **M. J. Bulger**, M.D.Dub., B.Ch., District Medical Officer of the Islington Parish. **Peter Campbell**, M.B., Certifying Factory Surgeon for the Newport District, Fifeshire. **Wm. J. Brereton Fergus**, M.B., B.Ch., B.A.O.R.U.I., Assistant House-Surgeon and Visiting Surgeon to the Stockport Infirmary. **G. L. Godwin**, L.S.A., District Medical Officer of the Worksop Union. **T. T. Harratt**, M.R.C.S, L.R.C.P.Lond., Certifying Factory Surgeon for the Rye District, Sussex. **J. H. Hart**, M.R.C.S., L.R.C.P., Assistant Medical Officer of the Wandsworth and Clapham Union Infirmary. **T. Howard**, M.B., Certifying Factory Surgeon for the Portland District, Dorset. **J. R. Hutchinson**, M.B., Ch.B.Vict., Resident Assistant Medical Officer of the Chorlton Union. **T. C. Lawson**, M.R.C.S.Eng., District Medical Officer of the Bourne Union. **Arthur W. Lemarchand**, M.R.C.S., L.R.C.P., Medical Referee under the Workmen's Compensation Act for Barnstaple, Bideford, South Molton, and Torrington in County Court Circuit, No. 57. **J McClintock**, L.R.C.P.&S.Edin., District Medical Officer of the Church Stretton Union. **J. McLaughlin**, M.D.(R.P.), Lieutenant-Colonel R.A.M.C., Recruiting Medical Officer at Bradford. **R. L. Roberts**, M.D.Lond., M.R.C.S., Certifying Factory Surgeon for the Ruabon District, Denbighshire. **T. M. Tibbetts**, M.B.Lond., M.R.C.S., District Medical Officer of the Dudley Union. **Arthur W. Tuxford**, M.B., L.R.C.P., M.R.C.S., D.P.H., Resident Medical Officer to the Baguley Sanatorium, near Manchester.

**"The Hospital" Institutional Library.**

"Hospitals and Asylums of the World." 4 Vols., with a Portfolio of Plans, complete £12 12s.

"Burdett's Hospitals and Charities, 1903." 5s. net.

"Burdett's Official Nursing Directory, 1903." 3s. net.

"Cottage Hospitals, General, Fever, and Convalescent." 10s. 6d.

"Uniform System of Accounts for Hospitals and Public Institutions." New Edition. 4s. net.

"Hospital Expenditure: The Commissariat." 2s. 6d.

"A Legal Handbook for the Use of Hospital Authorities." 2s. 6d.

"The Cottage Hospital Case Book or Register of Patients." 10s. and 12s. 6d.

All these are published by the Scientific Press, Ltd., and may be obtained through any bookseller, or direct from the publishers, 28 and 29 Southampton Street, Strand, London, W.C.

ST. BARTHOLOMEW'S HOSPITAL AND COLLEGE.

The WINTER SESSION will begin on Thursday, October 1st, 1903.

Students can reside in the College within the Hospital walls, subject to the Collegiate regulations.

The Hospital contains a service of 750 beds. Scholarships and Prizes of the aggregate value of nearly £800 are awarded annually. The Medical School contains large Lecture Rooms and well-appointed Laboratories for Practical Teaching, as well as Dissecting Rooms, Museum, Library, &c.

The Amalgamated Clubs' Ground (10 acres) is at Winchmore Hill, within easy reach of the Hospital.

For further particulars apply, personally or by letter, to the Warden of the College, St. Bartholomew's Hospital, E.C.

A Handbook forwarded on application.

### Valuable Text-Books for Nurses.

### A HANDBOOK FOR NURSES.

By Dr. J. WATSON.

Price 5/- net, 5/4 post free.

### NURSING: ITS THEORY AND PRACTICE.

By Dr. PERCY LEWIS.

Price 3/6 post free.

### NURSING.

By ISABEL HAMPTON.

Formerly Matron of the Johns Hopkins Hospital, Baltimore.

Price 7/6 net, 7/10 post free.

### SURGICAL WARD-WORK AND NURSING.

By Dr. A. MILES.

Price 3/6 net, 3/10 post free.

### THE HUMAN BODY: Practical Physiology and Hygiene.

By Professor COLTON.

Price 5/- post free.

### Pocket Books for Nurses.

### THE NURSES' DICTIONARY OF MEDICAL TERMS.

By HONNOR MORTEN.

Price 2/- post free.

### PRACTICAL GUIDE TO SURGICAL BANDAGING AND DRESSINGS.

By W. JOHNSON SMITH, F.R.C.S.

Price 2/- post free.

*Obtainable of all Booksellers, and published by*
THE SCIENTIFIC PRESS, LIMITED, 28 & 29 Southampton Street, Strand, London,
*who will send their latest Catalogue of Nursing Text-books post free to any Nurse on application.*

